# An Immunohistochemical Study of Gastric Mucosa and Critical Review Indicate That the Subepithelial Telocytes Are Prelymphatic Endothelial Cells

**DOI:** 10.3390/medicina55070316

**Published:** 2019-06-27

**Authors:** Oana D. Toader, Mugurel C. Rusu, Laurenţiu Mogoantă, Sorin Hostiuc, Adelina Maria Jianu, Adrian Cosmin Ilie

**Affiliations:** 1 Department XIII of Obstetrics, Gynecology and Neonatology, “Polizu” Clinical Hospital, “Carol Davila” University of Medicine and Pharmacy, 020021 Bucharest, Romania; 2 Division of Anatomy, Faculty of Dental Medicine, “Carol Davila” University of Medicine and Pharmacy, 020021 Bucharest, Romania; 3 Department of Histology, University of Medicine and Pharmacy Craiova, 2 Petru Rares Street, 200349 Craiova, Dolj, Romania; 4 Department of Legal Medicine and Bioethics, Faculty of Dental Medicine, “Carol Davila” University of Medicine and Pharmacy, 020021 Bucharest, Romania; 5 Division of Anatomy, “Victor Babeş” University of Medicine and Pharmacy, 300041 Timişoara, Romania

**Keywords:** lymphatic endothelial cell, mesenchymal cell, intestinal stem niche, lamina propria, pericryptal fibroblasts

## Abstract

*Background and Objectives:* There are only a few studies regarding gut subepithelial telocytes (TCs). The telopodes, namely peculiar TCs’ prolongations described on two-dimensional cuts, are not enough to differentiate this specific cell type. Subepithelial TCs were associated with the intestinal stem niche but a proper differential diagnosis with lymphatic endothelial cells (LECs) was not performed. In this study, we will also critically review studies suggesting that distinctive TCs could be positioned within the lamina propria. *Materials and Methods:* We performed an immunohistochemical study of human gastric mucosa to test the expression of D2-40, the lymphatic marker, as well as that of CD31, CD34, CD44, CD117/c-kit, α-smooth muscle actin (α-SMA) and vimentin in the gastric subepithelial niche. *Results:* The results support the poorly investigated anatomy of intramural gastric lymphatics, with circumferential collectors located on both sides of the muscularis mucosae (mucosal and then submucosal) and myenteric collectors in the muscularis propria. We also found superficial epithelial prelymphatic channels bordered by D2-40+ but CD31–TC-like cells. Deep epithelial lymphatic collectors drain in collectors within the lamina propria. Blood endothelial cells expressed CD31, CD34, CD44, and vimentin. *Conclusions:* Therefore, the positive diagnosis of TC for subepithelial CD34+ cells should be regarded with caution, as they could also be artefacts, resulting from the two-dimensional examination of three dimensional structures, or as LECs. Lymphatic markers should be routinely used to discriminate TCs from LECs.

## 1. Introduction

Telocytes is the name given to interstitial cells with small cellular bodies, bearing prolongations (telopodes) that are extremely thin, long and moniliform [1]. It has been emphasised that the telopodial emergence and the size of the cell body and telopodes should differentiate TCs from various types of fibroblasts [1]. In TCs, cell prolongations directly emerge from the cell body (the telopodial emergence) and should be differentiated from hybrid morphologies or from a thick emergence of processes from the cell body, followed by a gradual thinning [1]. It has repeatedly been indicated that telopodes alone are not enough to distinguish TCs [1,2]. On two-dimensional cuts, the morphological appearance of TCs could be caused by tangential cuts of endothelial tubes. In tissues where TCs were previously identified using transmission electron microscopy (TEM), three-dimensional ultrastructural studies demonstrated that TCs do not exist and, in fact, in single plane, the thin, flat cell body of pancake-like cells might occasionally be misinterpreted as telopodes [3]. The TCs identification by immunohistochemistry is still uncertain. Additionally, TCs do not appear in the accepted Terminologia Histologica, and considerable artifactual results that have been used to support TCs as a novel cell type, are extensively documented [4,5,6,7]. Moreover, recent reviews suggest that TCs have not been adequately distinguished from lymphatic endothelial cells (LECs) in previous studies [5], which makes their morphological identification as well as their repeatedly claimed CD34+ immunophenotype [8,9,10,11,12] insufficient for a firm assessment. Diaz-Flores et al. (2014) proposed an algorithm to distinguish the CD34+ stromal cells/TCs from endothelial cells: The stromal cells/TCs could be identified by a CD34+, CD31-, α-SMA- phenotype, the blood endothelial cells would be identified by a CD34+, CD31+, α-SMA- phenotype, while LECs would express CD31 but not CD34 and α-SMA [13]. However, few recent studies suggest that LECs could also express CD34 [14,15], contrary to a previous one that found LECs expressing LYVE-1, a lymphatic cell marker, but having a negative expression of PAL-E and CD34 [16]. A different protocol for isolating normal and diseased LECs by multi-parameter fluorescence-activated cell sorting rejected the use of LYVE-1 [17] because that marker was found strongly expressed in the initial lymphatics but its expression was either very weak, or absent, in the lymphatic collecting vessels [18]. The lymphatic endothelial cell phenotype was validated by the immunohistochemical detection of the following markers: PROX-1, VEGFR-3, Podoplanin, CD34 and CD31 when the cells were first split for further expansion [17]. In normal LECs was found a CD34^Low^CD31^Pos^VEGFR-3^Pos^PODOPLANIN^Pos^ phenotype but the diseased LECs had a CD34^High^CD31^Pos^VEGFR-3^Pos^PODOPLANIN^Pos^ phenotype [17]. Therefore, the use of CD34 and LYVE-1 in discriminating blood endothelial cells from LECs, or LECs from TCs, should be considered with caution. Nevertheless, Diaz-Flores et al. (2014) discussed that CD34+ stromal cells or TCs “behave as native mesenchymal stem cell progenitors after losing CD34 expression”, and therefore the expression of CD34 seems to be not mandatory in cells with telopodes.

A recently published paper in Nature by Shoshkes-Carmel et al. has identified ‘subepithelial telocytes’ (SETCs) as a source of Wnt proteins (Wnt2b, Wnt5a) that support the intestinal stem niche [19]. Similar to Wnt5b, Wnts play key roles in vascular morphogenesis, vessels sprouting and elongation, but also in the development of lymphatic capillaries [20,21,22]. These SETCs have been introduced as ‘large but rare mesenchymal cells’, forming a subepithelial plexus from the stomach to the colon and expressing FOXL1, PDGFRα and CD34 [19]. In the lung, PDGFRα-expressing stroma cells also express Wnt2, Wnt2b, Wnt9b and Wnt5a [23]. The SETCs description as ‘large’ and ‘mesenchymal’ cells is different from the ‘small’ and ‘interstitial’ TCs and could cause confusion. Moreover, a single figure depicts such a SETC in transmission electron microscopy with such a small prolongation that can hardly be taken as a telopode [19]. Another recent paper speculated that the CD34+ spindle-shaped cells with long process cells are TCs, and detected subepithelial CD34+ TCs in rat prostate [8]. Previously, subepithelial TCs were also found in the parotid gland; however, they were not tested for the expression of CD34 [24]. Neither was the term ‘lymphatic’ found in papers identifying subepithelial CD34+ TCs [8,19], nor were specific LECs markers such as podoplanin or LYVE-1 used to discriminate TCs from LECs [25]. This is a serious problem, as, in two-dimensional tangential cuts, LECs could appear as TC-like cells [26], hence the possibility of easily challenging the immunohistochemistry proofs.

We therefore intended to perform an immunohistochemical study using CD34 and podoplanin on human gastric wall samples, to critically review the results and document the publications identifying SETCs in the gut.

## 2. Material and Methods

The immunohistochemical study was performed retrospectively on archived paraffin-embedded samples of human stomach (ten cases). The ages of donor patients ranged from 56 to 62 years. The patients’ signed informed consent for all medical data to be used for research purposes, provided that the protection of their identity is maintained, foreran tissues processing. The study was tacitly approved by the responsible authorities where the work was carried out, and it was conducted in accordance with the general principles of medical research, as stated in the Declaration of Helsinki. All the procedures were followed by the Institutional Ethics Committee of the University of Medicine and Pharmacy Craiova (ref. no. 74/11.07.2016).

The paraffin-embedded samples were processed with an automatic histoprocessor (Diapath, Martinengo, BG, Italy). Sections were cut at 3 μm and mounted on SuperFrost^®^ electrostatic slides for immunohistochemistry (Thermo Scientific, Menzel-Gläser, Braunschweig, Germany). Histological evaluations used 3 μm-thick sections stained with hematoxylin and eosin. Internal negative controls resulted when the primary antibodies were not applied on slides.

We used primary antibodies for CD34 (Cat# CM 084 A, B, C, clone QBEnd/10, Biocare Medical, Concord, CA, USA, 1:50), D2-40 (Cat# CM 266 A, B, C, clone D2-40, Biocare Medical, Concord, CA, USA, 1:100), alpha-smooth muscle actin (Cat# CM 001 A, B, C, clone 1A4, Biocare Medical, Concord, CA, USA, 1:50), CD117/c-kit (Cat# CME 296 CK, RRID:AB_10581361, clone Y145, Biocare Medical, Concord, CA, USA, 1:100), CD31 (PECAM-1) (Cat# CM 347 A, C, clone BC2, Biocare Medical, Concord, CA, USA, 1:200), CD44 (Cat# CM 318 A, RRID:AB_10583032, clone 156-3C11, Biocare Medical, Concord, CA, USA, 1:150) and vimentin (Cat# CRM 312 A, B, clone SP20, Biocare Medical, Concord, CA, USA, 1:100). 

Tissues were deparaffinized and rehydrated; then endogenous peroxidase was blocked using Peroxidazed 1 (Biocare Medical, Concord, CA, USA). For the heat-induced epitope retrieval, we used the Decloaking Chamber (Biocare Medical, Concord, CA, USA) and retrieval solution pH 6 (Biocare Medical, Concord, CA, USA). We used Background Blocker (Biocare Medical, Concord, CA, USA) to reduce nonspecific background staining. The primary antibody was then applied. We used different HRP-based detection systems: For CD34 the two-steps detection used a 4 plus detection system, for α-SMA, CD44 and D2-40 we used MACH 4 (Biocare Medical, Concord, CA, USA) and for vimentin, CD31 and CD117 we used MACH 2 (Biocare Medical, Concord, CA, USA). A HRP-compatible chromogen (DAB) was applied. Sections were counterstained with hematoxylin and rinsed with deionized water. For the washing steps, we used TBS solution, pH 7.6.

## 3. Results

### 3.1. Gastric Histology Was Accurately Recognised

On the HE-stained slides, we properly differentiated the general histology of the gastric wall, which consisted of an inner mucosa, an outer submucosa, the muscularis externa and serosa. The gastric mucosa was built up by the gastric epithelium, containing the gastric glands and the lamina propria, subsequently appearing as bi-layered: The connective/stromal layer was facing the epithelium and the muscularis mucosae was facing the submucosa. 

### 3.2. Subepithelial Telocytes (TCs) Are a Mixture of Blood and Lymphatic Endothelial Cells

Within the gastric mucosa, we found CD117/c-kit-expressing mast cells. No other cell types were found expressing that marker.

**Figure 1:**
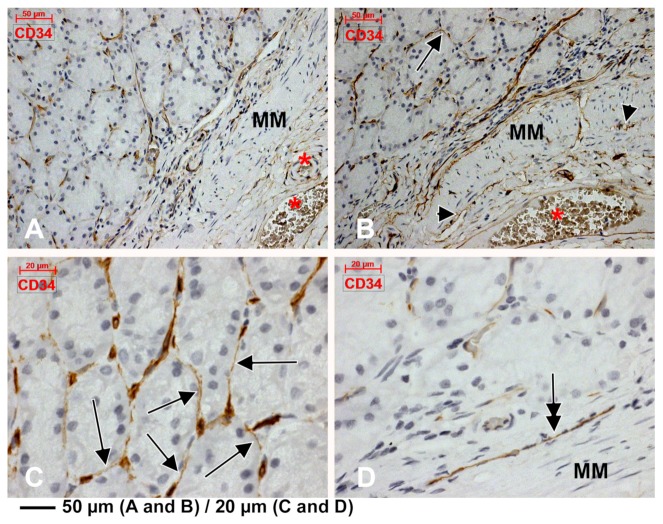
Immunoexpression of CD34 in the gastric mucosa. Endothelial tubes immediately beneath the glandular epithelium are found and their tangential cuts display a telocyte (TC)-like morphology on two-dimensional cuts (**B**,**C**, arrows). Larger blood vessels are found within the lamina propria (**A**), adluminally to the muscularis mucosae (MM). Thin CD34+ endothelial conduits lacking blood cells are applied on the MM (B, arrowheads). A CD34+ endothelial cord is presented (**D**, double-headed arrow). Large submucosa blood vessels are found abluminally to the MM (**A**,**B**, *).

**Figure 2:**
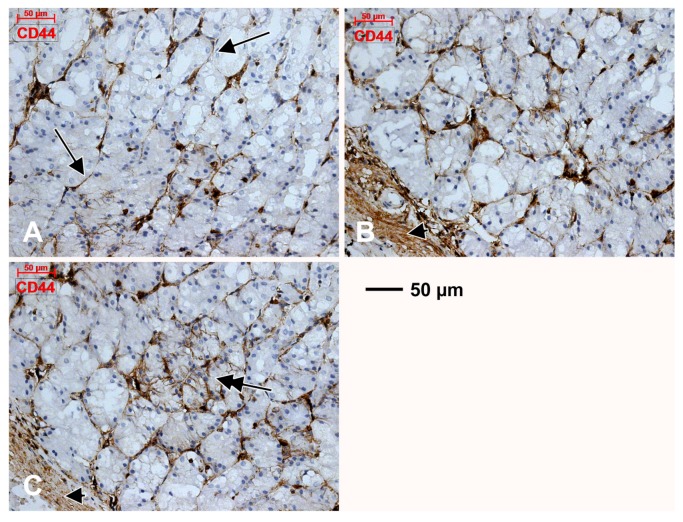
Within the gastric mucosa CD44 is expressed in endothelial tubes and TC-like tangentially cut endothelial cells beneath the glandular epithelium (**A**, arrows), as well as in the muscularis mucosae (**B**,**C**, arrowheads). The patchy expression of CD44 in glandular epithelia is also found (**C**, double-headed arrow).

**Figure 3:**
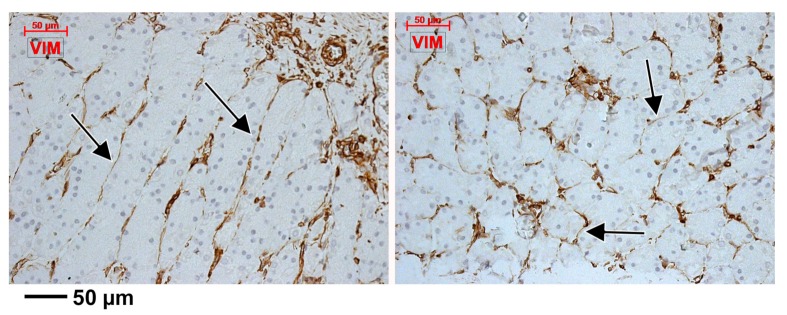
Expression of vimentin is pan-stromal in the gastric mucosa, including the subepithelial endothelial tubes and TC-like endothelial cells (arrows).

Just beneath the mucosal layer, we found subepithelial spindle-shaped TC-like cells, on the two-dimensional slices in the periglandular stroma of gastric mucosa. They uniformly expressed CD34, CD44 and vimentin (Figure 1, Figure 2 and Figure 3). These markers were also expressed on blood endothelial cells and thin-walled endothelial tubes not filled with RBCs, which were assessed as being lymphatic. We detected a patchy positive glandular epithelial expression of CD44 (Figure 2). 

**Figure 4:**
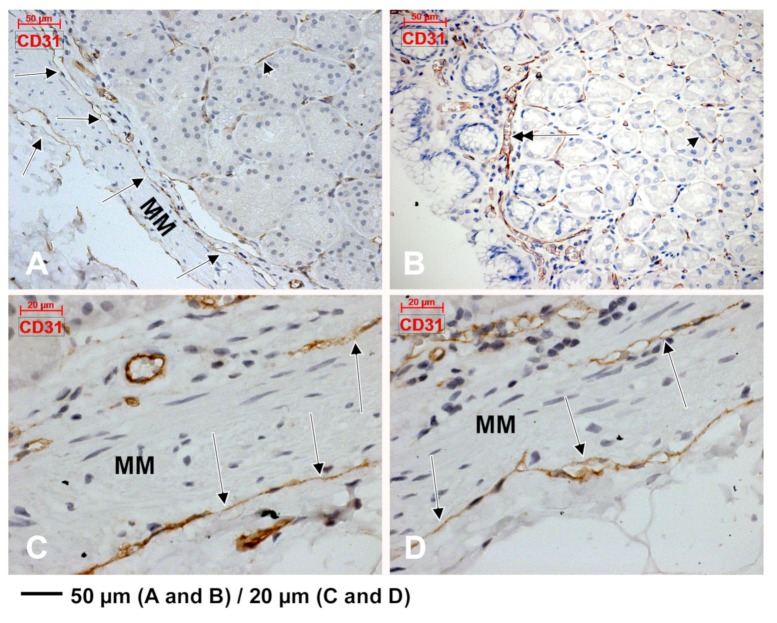
Immunoexpression of CD31 in the gastric mucosa is found on both sides of the muscularis mucosae (MM) in thin-walled lymphatic vessels free of blood cells (**A**,**C**,**D**, arrows). Beneath glandular epithelia the endothelial expression of CD31 is scarce (**A**,**B**, arrowheads). A superficial capillary is found beneath the surface epithelium (**B**, double-headed arrow).

**Figure 5:**
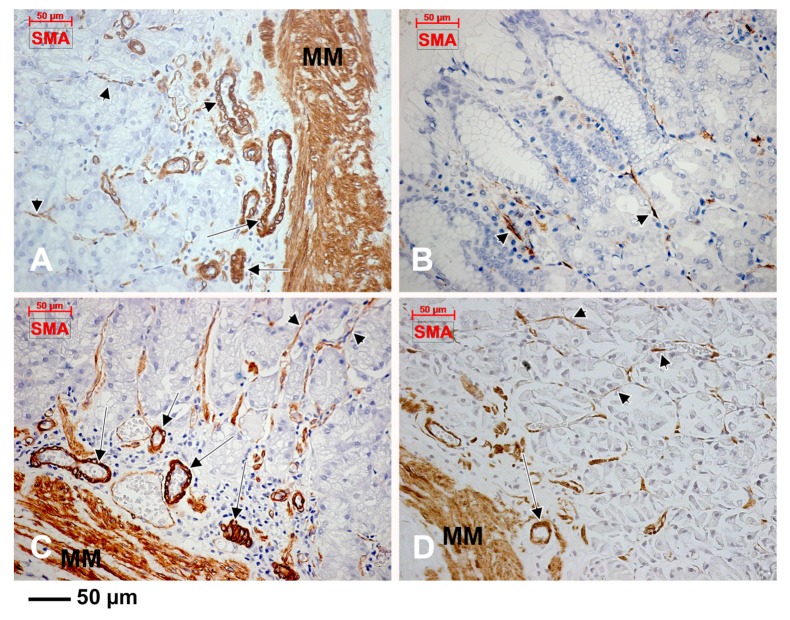
The expression of α-smooth muscle actin (α-SMA) is encountered in the muscularis mucosae (MM), vascular smooth muscle cells (**A**,**C**,**D**, arrows) and pericytes (**A**–**D**, arrowheads).

**Figure 6:**
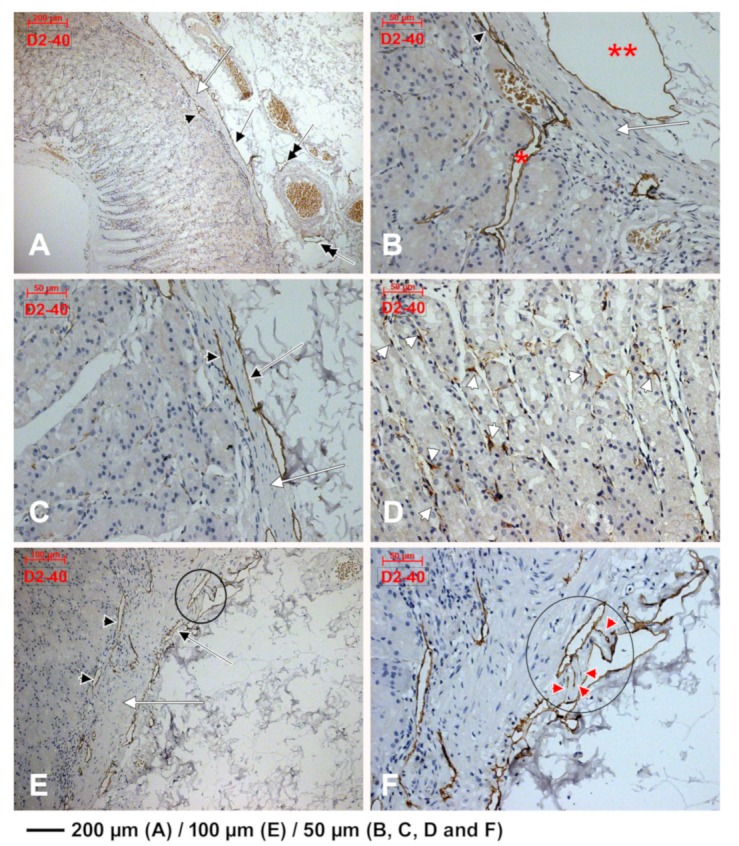
D2-40 expression identifies lymphatic plexuses applied on both sides of muscularis mucosae (**A**–**C**,**E,** white arrow). The mucosal lymphatic plexus (**A**–**C**,**E**, black arrowheads) lies within the deep lamina propria and receives collectors from the deep epithelial layer (**B**, *). The submucosal lymphatic plexus lies beneath the muscularis mucosae, has large collectors (**B**, **) and blind ends (inset in (**E**), detailed at higher magnification in (**F**), red arrowheads). These lymphatic collectors, if tangentially cut, display a false TC-like morphology (**B**, black arrow). Prelymphatic subglandular spaces are incompletely limited by D2-40 spindle-shaped TC-like cells (**D**, white arrowheads).

CD31 was scarcely expressed in subepithelial endothelial tubes from the periglandular stroma (Figure 4). The myoid phenotype of microvessels’ mural cells, vascular smooth muscle cells and pericytes, were assessed using the anti-α-SMA antibody (Figure 5). D2-40 had a patchy expression in the subepithelial TC-like cells of the periglandular stroma (Figure 6). Using D2-40, we found that on both the epithelial and submucosal sides of the muscularis mucosae were interconnected circumferential lymphatic vessels of the mucosal and consequently the submucosal lymphatic plexuses (Figure 6), which also expressed CD31 (Figure 4). We found blind-ended capillaries in the lymphatic network of the submucosa (Figure 6). The mucosal lymphatic plexus drained the well-configured radially coursing lymphatic vessels of the deep epithelial layer, filled with prelymphatic interstitial spaces or initial lymphatics within the superficial epithelial layer, which were bordered discontinuously by the D2-40+ TC-like immediate subepithelial cells (Figure 6). Large lymphatic collectors were also found within the muscularis propria (the results have not been presented here).

We concluded that the CD31+, CD34+ and D2-40+ cords, masquerading TCs, were in fact endothelial, blood or lymphatic tubes, tangentially cut or completely compressed. Similarly, we concluded that the immediate subepithelial TC-like cells, labelled uniformly with CD34, CD44 and vimentin but patchy with CD31 and D2-40, are in fact cuts of endothelial cells, one subset being blood endothelial cells and another subset being lymphatic endothelial cells.

## 4. Discussion

The gut SETCs were found to have a FOXL1, PDGFRα and CD34 positive immunophenotype [19]. In the gastric wall we found two subsets of TC-like subepithelial cells on two-dimensional cuts: A general subset that expressed CD34, CD44 and vimentin and a patchy subset expressing CD31, α-SMA and D2-40. The presence and characteristics of these cells require an in depth discussion regarding their positive and differential diagnosis.

### 4.1. The Lymphatics of the Gastrointestinal Tract Were Poorly Documented

The intramural lymphatics of the gastrointestinal tract were usually overlooked in anatomical studies. There is a consistent lack of knowledge regarding the distribution of gastric lymphatics [27]. During the morphogenesis of the gastric intramural lymphatics in rats, are identifiable structures such as blind ends, lymphatic islands and connecting lymphatics [28]. In Japanese monkeys, the amount of lymphatic blind ends and branches increases in mature gastric walls, compared to the developing stages [29]. A few or no gastric lymphatics were found in the rat muscularis propria [28]. Several studies have patterned the intramural lymphatic anatomy of the gut (Table 1).

Initial lymphatic sinuses were located in the gastric mucosa near the pyloric glands [36], which correspond to the prelymphatic spaces we found inconsistently bordered by TC-like D2-40+ cells in the superficial epithelial layer of the gastric mucosa. A study on the human gastric samples used HE and toluidine blue stains and found no major differences in the microvasculature and lymphatics of the lamina propria, muscularis mucosae and the submucosa in different topographical locations (cardia, greater curvature, pyloric antrum) [37]. Similar to our study, lymph capillaries were seen in the submucosa and deep lamina propria adjacent to the muscularis mucosae [33,37]. Interestingly, when an anti-Factor VIII antibody was applied, it could not differentiate blood endothelial cells from LECs [37]. Therefore, although TC-like cells were found to contain Weibel-Palade bodies [2,38,39], the ultrastructural markers of an endothelial lineage, they could equally indicate blood capillaries and lymphatics. Epithelial prelymphatic spaces (initial lymphatics) and mucosal lymph capillaries, such as those we found by applying the D2-40 antibody, had not been observed previously in human samples, neither with histologic stains nor in transmission electron microscopy [37]. These results were subsequently supported by immunohistochemical and transmission electron microscopic studies, which found lymphatic networks on both sides of the muscularis mucosae; however, we also found some lymphatics associated with the base of the lowest gastric glands, a pattern described by other authors [33]. Listrom and Fenoglio-Preiser used only antibodies against laminin and Factor VIII [33] and have not described the prelymphatic epithelial spaces. Perhaps they could have been better observed by using specific lymphatic markers, such as podoplanin (Gp38, T1α, AGGRUS, D2–40), Prox-1 (Prospero-related homeobox transcription factor), VEGFR-3 (Flt-4) or LYVE-1 (Hyaluronan receptor) [40]. D2-40 is a useful marker for discriminating the invasion of lymphatic vessels from capillaries, venules and veins [41]. This is important in oncology, to indicate why the intramucosal gastric carcinoma has potential for lymph nodes metastasis; however, the intramucosal colonic carcinoma is biologically benign, although both the stomach and colon seemingly share a similar pattern of intramural lymphatic anatomy [33]. Our findings suggest that the epithelial prelymphatic spaces, which drain lymph through deep epithelial vessels towards the plexuses on both sides of the muscularis mucosae, justify the potential for lymph metastases of the intramucosal gastric carcinoma. Nevertheless, the pathological changes of gastric mucosa could better allow a malign infiltration into the lamina propria with direct dissemination through the lymphatic plexus on the adluminal side of the muscularis mucosae, as previously hypothesised [33]. 

### 4.2. Expression of CD44 in the Gastric Mucosa

It is important to discuss here the potential significance of the CD44+ expression in glandular epithelial cells that we found in the gastric mucosa, which is not uncommon [42], mainly because the CD44 expression is normally associated with vasculogenic structures and stemness [25]. The epithelial expression of CD44 suggests these cells to have acquired properties that may be characterized as increased stemness. This could be also true for the immediate subepithelial TC-like cells expressing CD44. It seems reasonable to hypothesize that epithelial-mesenchymal transformation (EMT) processes could recruit subepithelial cells, which could further gain a podoplanin+ phenotype. This is supported by the observation that EMT relies upon the loss of E-cadherin adhesion protein, which is functionally linked to the expression of podoplanin (E-cadherin is downregulated by podoplanin) [43]. Such EMT generates mesenchymal cells with stem properties, which could also express podoplanin before a functional commitment to lymphvasculogenesis.

### 4.3. Subepithelial ‘Telocytes’ Were Previously Studied as ‘Fibrocytes’ and as ‘Pericryptal Fibroblasts’

Cells with telopodial prolongations were found as early as 1964 in transmission electron microscopy in the gut lamina propria, beneath the glandular epithelium, termed at that time ‘fibrocytes’, forming ‘an almost complete layer’ enveloping the glands and larger vessels [44]. Lymphatic vessels were also found within the lamina propria [44].

Further, terminology was switched to pericryptal sheaths built-up by fibroblasts, regardless of the fact that they were previously introduced as ‘epithelioid connective tissue cells’, ‘penetrating fibroblasts’ or ‘pale cells’ by different authors, as reviewed in [45]. These ‘pericryptal fibroblasts’ (PFs) are self-renewing cells, which form a pericryptal sheath and migrate beneath the epithelium evolving from an undifferentiated phenotype to a differentiated one [45]. In transmission electron microscopy studies, these PFs also have telopodial prolongations [45,46]. However, no study on TCs to date discussed or differentiated TCs as a novel cell type from the gut-specific PFs. Interestingly, D2-40+ PFs were associated with various disorders [41]. Nevertheless, one should note the observation of Diaz-Flores et al. (2016) that CD34+ stromal fibroblastic/fibrocytic cells (CD34+ stromal cells, CD34+ fibroblasts, CD34+ fibrocytes, telocytes) are just “a subset of fibroblasts located in both perivascular and stromal positions in the connective tissue of multiple anatomical sites”. Diaz-Flores et al. quoted Barth and Westhoff, who previously observed that “probably, the authors were unaware of the fact that the cell population they investigated and described were identical” [47].

### 4.4. The Markers Used on Gut Subepithelial Cells Are Not Enough for a Positive Diagnosis of Telocytes

The FOXL1+ SETCs were also found expressing PDGFRα and CD34 [19], which are endothelial cell markers. This would not be improper as subsets of TCs were shown to have an endothelial phenotype [38], and TCs of the gastrointestinal tract expressed PDGFRα and CD34, similar to endothelial cells [10]. Moreover, TCs in the gut express SK3 [48], which can also be found in the endothelial cells. Therefore, the expression of PDGFRα in SETCs should be considered with caution. PDGFRα IHC is problematic; heterogeneous results in terms of staining specificity are obtained depending on the antibodies and methods used; moreover, the use of specific polyclonal antibodies might lead to non-specific detection of proteins [49]. Studies showing a PDGFRα+ for TCs in the GI tract used a marker from R & D Systems, Catalogue no. AF-307-NA, which we checked on the manufacturer’s website and found it to be a polyclonal antibody (https://www.rndsystems.com/products/human-pdgf-ralpha-antibody_af-307-na). As was recently observed, the CD34+/PDGFR-α+ phenotype of TCs resulted after cell cultures that did not use any sorting molecules, those cells being sorted solely based on morphological criteria [50].

A year before the appearance of the study regarding FOXL1+, PDGFRα+ and CD34+ SETCs [19], Stzepourginski et al. identified the pericryptal mesenchymal cells (crypt stromal cells, CSCs), apparently identical to the SETCs, which express CD34 and Gp38 (podoplanin) and are the major intestinal producers of the niche factors Wnt2b, Gremlin1 and R-spondin1 [51]. Stzepourginski et al. did not indicate in 2017 any cell type they studied as ‘telocyte’, although TCs were suggested being a distinctive cell type since 2010 [52]. These CD34+ Gp38+ CSCs acquire the characteristics of lymphoid stromal cells and initiate lymphoid tissue genesis, as these adhesion molecules are essential for the establishment of the cell-cell contact between stromal cells and lymphocytes [51]. Noteworthy, CD45-/CD90+/CD31+ intestinal LECs in the lamina propria produce R-spondin3, which is structurally similar to the other three members of the R-spondin family which potentiate Wnt/β-catenin signalling by inhibiting the turnover of Wnt receptors [53].

In liver, periportal FOXL1+ mesenchymal cells were characterised as progenitor cells [54]. Liver progenitor cells were also found to be positive for CD34 and Connexin 43 (Cx43) [55]. Although cultured TCs were shown to have a CD34+/vimentin+/Cx43+ phenotype [56], the authors did not discuss a possible lymphatic endothelial cells (LECs) lineage, although Cx43 and CD34 are expressed in LECs and have critical roles in lymphatic vascular development [57]. 

According to Shoshkes-Carmel et al., the SETCs are ‘an important source of Wnt proteins’ which influence the renewal of the epithelial niche [19]. However, the Wnt system is crucial in endothelial cell differentiation; therefore, the gut SETCs Wnt signalling could not be exclusively related to the epithelial niche. Moreover, shear stress (e.g. in the gut wall) regulates Wnt secretion from LECs [58].

### 4.5. Critical Mini-Review of the Studies Used to Assess Subepithelial Telocytes

The authors of the study on SETCs [19] quoted three previous studies on gut TCs [10,59,60], which allowed them to indicate the subepithelial mesenchymal cells as ‘telocytes’. A recent review consulted two of these telocytes-describing papers [59,60] and indicated that stromal signal-producing cells, ‘variously called telocytes and myofibroblasts’, express specific genes, such as PDGFRα and FOXL1 [23]. We carefully observed those three papers and gathered the elements (Table 2) which demand caution when referring to a subepithelial cell of the gastrointestinal tract as ‘telocyte’. It can be observed that two of these three published studies did not use specific markers and that one using markers did not use transmission electron microscopy.

**Figure 7:**
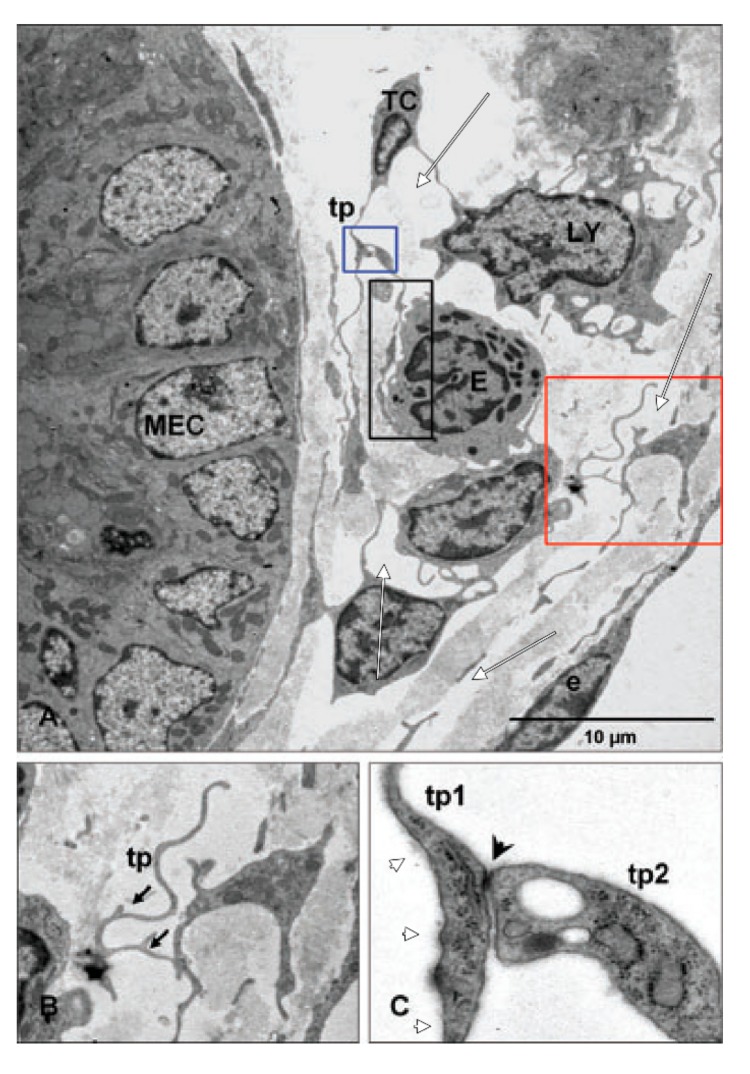
Reprinted with permission from John Wiley and Sons (License Number: 4447500883044) from [60]. The original legend of Figure 4 indicates: ”Relation of telocytes with other cells in the lamina propria. (**A**) The processes of a telocyte establish numerous interactions with adjacent cells in the lamina propria. A multicontact synapsis between an eosinophil and a telopode indicated by black rectangular area. Blue and red square illustrating higher magnification of telopodes (tp) from (**A**) in (**B**) and (**C**), respectively; (**B**) distinctive dichotomous pattern of branching (arrows); (**C**) direct cellular contact (arrowhead) between two telopodes (tp1, tp2) showing junctional complexes. MEC: Mucosal epithelial cell; LY: Lymphocyte; E: Eosinophile cells; e: Endothelial cell.”. We added white markups to indicate collagen-free lumina and interstitial spaces (white arrows) and anchoring filaments attached to telopodes (white arrowheads).

**Figure 8:**
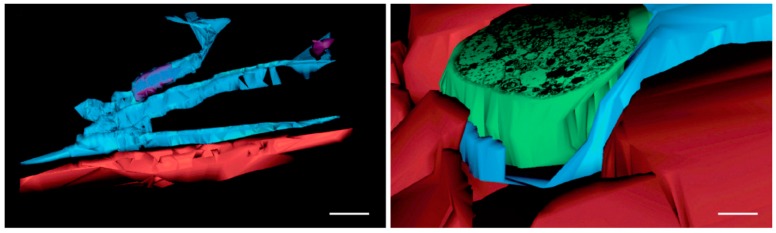
Reprinted with written permission from [59]. Cropped Figure 6 in the original article. The respective legend is “Figure 6. Rat jejunum. (A–E) 3D image reconstruction from 5 serial sections of TCs (blue) in lamina propria: Tp branching in a 3D pattern. TC nucleus is coloured in violet; (F–J) computer-aided volume rendering and different-angle stereoscopic views of a TC (blue) surrounding a nerve fiber (green) in muscularis mucosae (dark red). Scale bar: 2 μm”.

In one of these studies that we additionally reviewed here, it is stated that ‘TEM examination is fundamental in identifying the telocytes’ [60]. The authors introduced some cells as TCs, which were documented using low resolution micrographs. Apparently, those cells limit large interstitial spaces and large irregular lumina devoid of collagen fibres, lack basal lamina, overlap and have anchoring filaments (Figure 7) (e.g. in LECs). A second study found jejunal TCs ‘just beneath the epithelial layer of the mucosal crypts […]’ [59]—in the same location with the SETCs [19]. Interestingly, the digital reconstructions of the identified TCs depict thin, flat pancake-like cells rather than cell processes (Figure 8). Flat, pancake-like interstitial cells do not support the occurrence of TCs, as was recently proven [3]. The third paper identified submucosal ‘3-D networks’, built-up by PDGFRα+/CD34+ TCs but just on two-dimensional cuts and without the use of TEM [10]. These results were discussed in the previous section.

### 4.6. The Interstitial Organ: TC-Like Cells Limit Prelymphatic Spaces

Recently, Benias et al. argued in support of the concept of an interstitial organ widely distributed in human tissues. The authors’ study focused on the bile duct but was further sampled in the other tissues that are subject to compression (gastrointestinal tract, urinary bladder, dermis, peribronchial and periarterial tissues and fascia) [61]. The transmission electron microscopy sample of a fibroblast-like cell lining such as interstitial prelymphatic spaces was presented by these authors [61] and is similar to what numerous other papers have indicated as a ‘telopode’, as was recently observed [5]. The tissues that were subjected to intermittent or rhythmic compressions were already found containing cells with telopodes—the ICLCs/TCs [62,63,64,65,66]. Benias et al. offer convincing evidence that such interstitial cells that limit prelymphatic spaces tend to express CD34 and vimentin, often used as specific markers of ICLCs/TCs, as well as D2-40—a lymphatic marker [61]. The prelymphatic TC-like endothelial cells were found to be negative for CD117/c-kit [61], which also fits a CD117/c-kit- phenotype of ICLCs/TCs [64,67]. However, Benias et al. indicated that these prelymphatic endothelial cells do not express other lymphatic markers (e.g. LYVE-1) or CD31, known as a lymphendothelial marker [61]. We did not test the expression of LYVE-1. The ‘prelymphatic’ classification of the CD31-negative TC-like cells, bordering on one side the interstitial spaces, fits our findings: The scarce expression of CD31 but the general expression of D2-40 in the subglandular stroma with epithelial prelymphatics, which supply CD31+ and D2-40+ lymphatic plexuses in the lamina propria and submucosa. It appears that CD31 could discriminate between the prelymphatic TC-like cells, which do not express it, and the lymphendothelial cells, which express it. This has to be further validated, because studies that indicate that TCs do not express CD31 [13,67,68,69] did not test lymphatic markers. Although the negative expression of CD31 could exclude the discrimination of blood endothelial cells as TCs, it could hardly exclude the TC-like cells which limit prelymphatic spaces. However, although SETCs/pericryptal stromal cells can be distinguished as prelymphatic interstitial cells by the expression of podoplanin, the central lacteals may not be considered similar, although they express lymphatic markers. 

## 5. Conclusions

In conclusion, the gut subepithelial mesenchymal cells, usually introduced as pericryptal fibroblasts, should be treated with caution when being considered as ‘telocytes’. Cells similar to telocytes were observed early in the sixties, but the origin of these cells remains unclear. A subset of TCs seems to be CD34+ interstitial cells with endothelial features, which neighbour prelymphatic spaces and express podoplanin but do not express CD31. Lymphatic markers should be routinely used to discriminate TCs from LECs. A consensus should also be achieved between the terms ‘telocyte’ and ‘pericryptal fibroblasts’.

## Figures and Tables

**Table 1:** Lymphatics of the alimentary tract.

Organ	Intramural Lymphatics	Ref.
Oesophagus (human)	Lymphatic plexuses in the lamina propria, submucosa and muscularis propria	[30]
Stomach (rabbit)	Blind-ending lymphatic capillaries in the deep mucosa; layered lymphatic plexuses in the submucosa, muscularis propria and serosa	[31]
Stomach (Wistar rat)	Initial lymphatics in the mucosa, built-up by extremely thin endothelial cells. Submucosa, myenteric and serosal lymphatic networks.	[32]
Jejunum (Japanese monkey)	Central lymphatics in the villi are connected to an irregular mucosal lymphatic network consisting of tubular lymphatics. Tubulo-saccular lymphatics build a submucosa mesh-like network. Within the muscularis propria lies a maze-like lymphatic network. The lymphatic networks are independent of the blood vessels.	[33]
Small intestine (rat)	Lacteals in the villi fuse and form a wide sinus at the villous base which further drains into a submucosa lymphatic plexus. The coarse myenteric lymphatic plexus and the submucosa one drain through collecting lymphatics towards the mesenteric ones.	[34]
Large intestine (dog)	Superficial and deep lymphatic networks in the lamina propria, beneath the blood capillary networks. Thicker lymphatics and denser networks in the submucosa. Two efferent paths to the subserosa collecting trunks, a direct one and a minor one, the later supplemented by the lymphatics of the muscularis propria.	[35]

**Table 2:** Papers used to assess as ‘telocytes’ the FOXL1+ subepithelial mesenchymal cells [19].

Papers Which Support the Use of the Term ‘Telocytes’ for Subepithelial Mesenchymal Cells	TCs Identified in Light Microscopy (LM)	TCs Identified in Transmission Electron Microscopy (TEM)	TCs Were Differentiated from LECs
‘Identification of telocytes in the lamina propria of rat duodenum: transmission electron microscopy’ [60]	(+): LM on semithin sections	(+)	(–)
‘Telocytes, a distinct type of cell among the stromal cells present in the lamina propria of jejunum’ [59]	(+): LM on semithin sections	(+)	(–)
‘Telocytes express PDGFRa in the human gastrointestinal tract’ [10]	(+): IF, IHC	(–)	(–)

LM—light microscopy; TEM—transmission electron microscopy; LECs—Lymphatic Endothelial Cells; PDGFRa—Platelet-Derived Growth Factor Receptor a.

## Abbreviations

CSC	crypt stromal cell
Cx43	Connexin 43
FOXL1	winged helix transcriptional factor
LEC	lymphatic endothelial cell
PDGFR	platelet-derived growth factor receptor
PF	pericryptal fibroblast
SETC	subepithelial telocyte
TC	telocyte
TEM	transmission electron microscopy
